# Impact of age, sex and medical history on adverse reactions to the first and second dose of BNT162b2 mRNA COVID-19 vaccine in Japan: a cross-sectional study

**DOI:** 10.1186/s12879-022-07175-y

**Published:** 2022-02-23

**Authors:** Ryuta Urakawa, Emiko Tanaka Isomura, Kazuhide Matsunaga, Kazumi Kubota, Miho Ike

**Affiliations:** 1 Department of Pharmacy, Osaka University Dental Hospital, 1-8 Yamada-oka, Suita, Osaka 565-0871 Japan; 2 Department of Clinical Pharmacy Research and Education, Graduate School of Pharmaceutical Sciences, Osaka University, 1-6 Yamada-oka, Suita, Osaka 565-0871 Japan; 3 First Department of Oral and Maxillofacial Surgery, Graduate School of Dentistry, Osaka University, 1-8 Yamada-oka, Suita, Osaka 565-0871 Japan; 4 Second Department of Oral and Maxillofacial Surgery, Graduate School of Dentistry, Osaka University, 1-8 Yamada-oka, Suita, Osaka 565-0871 Japan; 5 Department of Healthcare Information Management, The University of Tokyo Hospital, 7-3-1 Hongo, Bunkyo-ku, Tokyo, 113-8655 Japan; 6 Nursing Department, Osaka University Dental Hospital, 1-8 Yamada-oka, Suita, Osaka 565-0871 Japan

**Keywords:** COVID-19, SARS-CoV-2, Vaccination, Adverse effect, Correlation

## Abstract

**Background:**

Vaccines for coronavirus disease 2019 (COVID-19) caused by severe acute respiratory syndrome coronavirus 2 (SARS-CoV-2) are being promoted worldwide. In this study, we analyzed the relationship between adverse reactions and the profile of vaccinated recipients.

**Methods:**

Vaccinated subjects who received two doses of BNT162b2 between May 17 and June 11, 2021, at Osaka University Dental Hospital were included in this study. Adverse reactions and profiles were collected by questionnaires, and the relationship between the presence of adverse reactions and the profiles of the vaccinated persons was analyzed by logistic regression analysis. The correlation between the severity of adverse reactions and age was analyzed by Spearman’s rank correlation.

**Results:**

Logistic regression analysis showed that, for many kinds of adverse reactions, the incidence was significantly higher in females than in males and in younger than in older people. There was a very weak but significant negative correlation between age and the severity of many kinds of adverse reactions. The relationship between sex and the incidence of each adverse reaction was significant for injection site reactions and fatigue in the first vaccination, whereas significant relationships were found for fatigue, chills, fever, arthralgia, myalgia and headache in the second vaccination, all of which were clearly more likely to occur in females.

**Conclusion:**

Adverse reactions to BNT162b2 were found to be more frequent and more intense in females and younger people in Japan, especially after the second vaccination.

## Introduction

The pandemic of coronavirus disease 2019 (COVID-19) caused by severe acute respiratory syndrome coronavirus 2 (SARS-CoV-2) is still mutating and spreading all over the world, leading to severe infections and deaths [1]. SARS-CoV-2 is transmitted by respiratory droplets, by aerosol particles, by direct contact with humans, or by indirect contact via contaminated objects [2–4]. COVID-19 causes various symptoms such as fever or chills, cough, shortness of breath or difficulty breathing, fatigue, muscle or body aches, headache, loss of taste or smell, sore throat, congestion or runny nose, nausea or vomiting, and diarrhea. In severe cases, death occurs mainly due to septic shock and multi-organ failure [5–7]. Because COVID-19 is infectious even before the onset of disease [8] and because a substantial proportion of infected people may be asymptomatic, infected people with no symptoms at all can be a potential source of infection [9].

While there are effective measures to prevent the spread of COVID-19, such as wearing masks, hand hygiene, and maintaining social distance [10–12], vaccination is a very effective way to prevent infection by acquiring antibodies against SARS-CoV-2. BioNTech started a first-in-human study of BNT162b1 (BNT162-01 trial) in Germany in April 2020 [13], and shortly thereafter Pfizer started a phase 1/2/3 study of BNT162b2 (C4591001 clinical trial) in the United States [14]. BNT162b2 was approved by the Medicines and Healthcare products Regulatory Agency (MHRA) in United Kingdom on December 2, 2020, and the world’s first vaccination with the licensed COVID-19 vaccine after clinical trials began on December 8, 2020. Since then, various vaccines against SARS-CoV-2 have been approved worldwide, including mRNA-1273 from Moderna/the Vaccine Research Center at the National Institute of Allergy and Infectious Diseases (NIAID), which is an mRNA vaccine similar to BNT162b2 [15]; viral vector vaccines such as ChAdOx1 from Oxford University/AstraZeneca [16] and Ad26.COV2.S from Johnson and Johnson [17]; and inactivated vaccines such as Sinovac’s CoronaVac [18]. Various other vaccines against SARS-CoV-2 have been approved and are being administered worldwide.

The COVID-19 vaccine is the world’s first mRNA-based vaccine deployed for primary prevention of infectious disease in humans, and its safety, efficacy and effectiveness are still being discussed by researchers. A recent study of self-reported adverse reactions to BNT162b2 in health care workers showed that although there are a variety of adverse reactions, most are not life-threatening and are well tolerated [19]. The results of other studies also supportsafety, efficacy and effectiveness [14, 20, 21]. However, data focusing on which vaccine recipients are most likely to have adverse reactions is limited and remains to be clarified.

Here, we hypothesized that the incidence and severity of BNT162b2 adverse reactions would vary depending on the profile of the vaccine recipient. In this study, we analyzed the profile of adverse reactions after vaccination and the profile of vaccine recipients using a self-reported adverse reaction questionnaire among vaccine recipients who received BNT162b2 at the Osaka University Dental Hospital.

## Methods

### Research subjects

All vaccinated hospital staffs who received the first dose of BNT162b2 between May 17 and May 21, 2021, and the second dose between June 7 and June 11, 2021, at the Osaka University Dental Hospital were included in this study. Among the subjects, those who did not submit the adverse reaction questionnaire and those who did not complete the confirmation of research consent column provided in the questionnaire were excluded from the study.

### Methods for investigating adverse reactions and vaccine recipient profiles

The questionnaire included columns for sex, age, study consent, current medical history, and presence and severity of adverse reactions. The questionnaire was anonymous. The current medical history included entries for heart disease, kidney disease, liver disease, blood disorders, blood coagulation system disorders, immunodeficiency, and others. The items of adverse reactions were injection site reaction, fatigue, chills, fever, arthralgia, myalgia, headache, diarrhea, nausea, and vomiting, and were prepared with reference to the severity of the Common Terminology Criteria for Adverse Events (CTCAE) Version 5.0. The results of the survey were collected from survey forms deposited in a collection box and from answers to a questionnaire posted on the Web.

### Statistical analysis

Adverse reactions after the first and second doses of vaccine were compared by Wilcoxon’s signed rank test. Univariate logistic regression analysis was performed with age or sex as independent variables and the presence or absence of various adverse reactions at the first and second time as dependent variables. Multivariate logistic regression analysis was then performed with age, sex, and previous medical history as covariates. In addition, Spearman’s rank correlation coefficient was performed to verify the relationship between age and the severity of each adverse reaction. All statistics were performed with IBM SPSS Statistics 26, and a two-tailed test with p < 0.05 was considered a significant difference.

### Ethical review

This study was conducted under the approval of the Ethical Review Committee of Osaka University Dental Hospital (Approval No. R3-E11).

## Results

827 hospital staffs (381 males and 446 females) received two doses of BNT162b2 vaccine. There were no staffs who received only one dose. A total of 460 vaccine recipients consented to the study and completed the questionnaire (participation rate: 55.6%). Of these responses, 458 questionnaires with no omissions were included in this study (valid response rate: 99.6%). The background of the study subjects is shown in Table 1. The results of Mann–Whitney U test showed that there was no statistically significant difference in age between males and females (p = 0.74).

**Table 1:** Background of the research subjects (N = 458)

Mean age (SD, range)	
All	38.9 (12.8, 19–77)
Sex	
Male number (%)	195 (42.6)
Female number (%)	263 (57.4)
Medical history	
Yes (%)	54 (11.8)
No (%)	404 (88.2)

Medical history was identified as heart disease, kidney disease, liver disease, blood disorders, blood coagulation system disorders, immunodeficiency, and others in this study

A comparison of adverse reactions after the first and second doses is shown in Table 2. There was no significant difference in diarrhea, but there were significant differences in other adverse reactions (i.e., injection site reaction, fatigue, chills, fever, arthralgia, myalgia, headache, and nausea) between the first and second doses.

**Table 2:** Severity of adverse reactions after the first and second vaccinations

Severity	Grade 0	Grade 1	Grade 2	Grade 3	p value
	First dose/second dose	First dose/second dose	First dose/second dose	First dose/second dose	
	n (%)	n (%)	n (%)	n (%)	
Injection site reaction	102 (22.3)/168 (36.7)	231 (50.4)/190 (41.5)	118 (25.8)/95 (20.7)	7 (1.5)/5 (1.1)	< 0.01
Fatigue	287 (62.7)/201 (43.9)	120 (26.2)/90 (19.7)	46 (10.0)/139(30.3)	5 (1.1)/28 (6.1)	< 0.01
Chills	447 (97.6)/389 (84.9)	9 (2.0)/48 (10.5)	2 (0.4)/21 (4.6)	0/0	< 0.01
Fever	446 (97.4)/346 (75.5)	12 (2.6)/102 (22.3)	0/10 (2.2)	0/0	< 0.01
Arthralgia	339 (87.1)/318 (69.4)	39 (8.5)/67 (14.6)	16 (3.5)/61 (13.3)	4 (0.9)/12 (2.6)	< 0.01
Myalgia	391 (85.4)/361 (78.8)	39 (8.5)/48 (10.5)	25 (5.5)/41 (9.0)	3 (0.7)/8 (1.7)	0.04
Headache	366 (79.9)/266 (58.1)	52 (11.4)/79 (17.2)	36 (7.9)/90 (19.7)	4 (0.9)/23 (5.0)	< 0.01
Diarrhea	455 (97.2)/439 (95.9)	11 (2.4)/16 (3.5)	0/2 (0.4)	2 (0.4)/1 (0.2)	0.35
Nausea	433 (94.5)/407 (88.9)	18 (3.9)/23 (5.0)	7 (1.5)/27 (5.9)	0/1 (0.2)	< 0.01
Vomiting	453 (98.9)/447 (97.6)	3 (0.7)/9 (2.0)	2 (0.4)/1 (0.2)	0/1 (0.2)	0.22

Values are number of adverse effect after first vaccination/second vaccination. Grade is the severity of each adverse reaction with reference to CTCAE Version 5.0. Chi-square test was performed to examine the difference between the first and second adverse reactions

The results of the univariate logistic regression analysis of the association between age or sex and the presence of adverse reactions are shown in Table 3. The results of multivariate logistic regression analysis with age, sex, and previous medical history as covariates are shown in Table 4. For many adverse reactions, females were found to have a significantly higher incidence of adverse reactions than males. Similarly, the incidence of many adverse reactions was found to be significantly higher in younger people. After adjusting for age, sex, and history, injection site reaction, chills, arthralgia, headache, and nausea were significantly more likely to occur in younger participants (odds ratio (OR) 0.72, 0.31, 0.77, 0.76, and 0.64, respectively) and injection site reaction and fatigue were significantly more likely to occur in females (OR 1.71 and 1.57, respectively) after the first dose. Similarly, after the second dose, injection site reaction, fatigue, chills, fever, arthralgia, myalgia, headache, diarrhea, and nausea were significantly more likely to occur in younger patients (OR 0.85, 0.83, 0.76, 0.74, 0.82, 0.77, 0.77, 0.47, and 0.70, respectively), and fatigue, chills, fever, arthralgia, myalgia, and headache were significantly more likely to occur in females (OR 1.74, 2.34, 1.79, 1.88, 1.88, and 2.27, respectively).

**Table 3:** Effect of age or sex on the presence of each adverse reaction

	Age		Sex	
	First vaccination	Second vaccination	First vaccination	Second vaccination
	OR (95%CI)	OR (95%CI)	OR (95%CI)	OR (95%CI)
Injection site reaction	0.75 (0.63–0.88)**	0.85 (0.73–0.98)*	1.72 (1.10–2.67)*	1.38 (0.94–2.03)
Fatigue	0.90 (0.78–1.05)	0.84 (0.73–0.97)*	1.58 (1.07–2.33)*	1.75 (1.20–2.55)**
Chills	0.30 (0.12–0.76)*	0.76 (0.61–0.95)*	0.41 (0.12–1.44)	2.37 (1.33–4.20)**
Fever	0.52 (0.28–0.98)*	0.75 (0.62–0.90)**	0.36 (0.11–1.22)	1.80 (1.15–2.81)*
Arthralgia	0.80 (0.63–1.01)	0.83 (0.71–0.98)*	1.01 (0.58–1.76)	1.88 (1.24–2.86)**
Myalgia	0.86 (0.70–1.07)	0.79 (0.65–0.95)*	1.39 (0.81–2.38)	1.88 (1.17–3.03)**
Headache	0.77 (0.63–0.93)**	0.79 (0.68–0.92)**	1.60 (0.99–2.58)	2.27 (1.54–3.34)**
Diarrhea	0.77 (0.47–1.25)	0.55 (0.33–0.89)*	0.86 (0.28–2.60)	0.82 (0.33–2.05)
Nausea	0.66 (0.45–0.97)*	0.73 (0.56–0.94)*	1.34 (0.58–3.10)	1.55 (0.84–2.87)
Vomiting	0.54 (0.21–1.40)	0.82 (0.49–1.36)	1.11 (0.18–6.73)	1.31 (0.38–4.52)
Any adverse event	0.69 (0.56–0.85)**	0.64 (0.52–0.80)**	2.13 (1.20–3.75)**	2.15 (1.21–3.83)**

Logistic regression was performed. *OR* odds ratio, *CI* confidence interval. *p < 0.05, **p < 0.01

OR and 95% CI of age show ratio per 10 years. OR and 95% CI of Sex show ratio of female to male

**Table 4:** Effect of age, sex, and medical history on the presence of each adverse reaction

	Age		Sex		Medical history	
	First vaccination	Second vaccination	First vaccination	Second vaccination	First vaccination	Second vaccination
	OR (95% CI)	OR (95% CI)	OR (95% CI)	OR (95% CI)	OR (95% CI)	OR (95% CI)
Injection site reaction	0.72 (0.60–0.86)**	0.85 (0.72–0.99)*	1.71 (1.09–2.68)*	1.36 (0.93–2.01)	1.86 (0.86–4.00)	1.13 (0.61–2.11)
Fatigue	0.90 (0.77–1.05)	0.83 (0.71–0.97)*	1.57 (1.06–2.32)*	1.74 (1.19–2.54)**	1.17 (0.63–2.17)	1.29 (0.70–2.38)
Chills	0.31 (0.12–0.82)*	0.76 (0.60–0.97)*	0.41 (0.12–1.45)	2.34 (1.32–4.17)**	N/A	0.94 (0.37–2.39)
Fever	0.57 (0.30–1.09)	0.74 (0.61–0.89)**	0.35 (0.10–1.20)	1.79 (1.13–2.82)*	N/A	1.27 (0.61–2.61)
Arthralgia	0.77 (0.60–0.98)*	0.82 (0.69–0.97)*	1.00 (0.57–1.75)	1.88 (1.24–2.87)**	1.62 (0.69–3.82)	1.40 (0.73–2.69)
Myalgia	0.84 (0.67–1.05)	0.77 (0.63–0.94)**	1.39 (0.81–2.39)	1.88 (1.16–3.04)*	1.50 (0.67–3.36)	1.51 (0.73–3.15)
Headache	0.76 (0.62–0.93)**	0.77 (0.66–0.91)**	1.58 (0.98–2.57)	2.27 (1.53–3.37)**	1.25 (0.58–2.71)	1.37 (0.73–2.57)
Diarrhea	0.78 (0.47–1.28)	0.47 (0.28–0.79)**	0.85 (0.28–2.56)	0.80 (0.31–2.03)	0.81 (0.10–6.69)	4.41 (1.26–15.43)*
Nausea	0.64 (0.43–0.95)*	0.70 (0.54–0.92)*	1.33 (0.57–3.09)	1.54 (0.83–2.87)	1.64 (0.45–6.03)	1.49 (0.57–3.86)
Vomiting	0.57 (0.22–1.52)	0.77 (0.45–1.31)	1.11 (0.18–6.74)	1.31 (0.38–4.54)	N/A	2.29 (0.44–11.94)
Any adverse event	0.67 (0.53–0.83)**	0.62 (0.49–0.78)**	2.10 (1.17–3.75)*	2.11 (1.16–3.82)*	2.16 (0.81–5.77)	2.30 (0.85–6.22)

Logistic regression was performed. *OR* odds ratio, *CI* confidence interval. *p < 0.05, **< p < 0.01

OR and 95% CI of age show ratio per 10 years. OR and 95% CI of sex show ratio of female to male. OR and 95% CI of medical history show ratio of those with a medical history to those without. All the statistics were adjusted for age, sex, and medical history

The correlation between age and the severity of each adverse reaction is shown in Table 5. Weak but significant negative correlations with age were detected for injection site reaction, chills, fever, arthralgia, and headache after the first vaccination and for injection site reaction, fatigue, fever, arthralgia, myalgia, headache, diarrhea, and nausea after the second vaccination.

**Table 5:** Correlation between age (years) and severity of adverse reaction

	First vaccination	Second vaccination
Injection site reaction	− 0.238**	− 0.163**
Fatigue	− 0.076	− 0.108*
Chills	− 0.140**	− 0.091
Fever	− 0.105*	− 0.134**
Arthralgia	− 0.108*	− 0.129**
Myalgia	− 0.077	− 0.130**
Headache	− 0.132**	− 0.143**
Diarrhea	− 0.053	− 0.130**
Nausea	− 0.087	− 0.117*
Vomiting	− 0.060	− 0.034

Correlation was measured by Spearman’s rank correlation. The values indicate the correlation coefficient. *p < 0.05, **< p < 0.01

## Discussion

Various types of vaccines against COVID-19 have been developed and are being administered around the world. In Japan, Pfizer’s BNT162b2 was approved in February 2021, and vaccinations began for healthcare workers. Subsequently, mRNA-1273 and AZD1222 were approved in May 2021 and, together with BNT162b2, have been promoted for vaccination of both healthcare workers and the public. In Japan, the initial target age group for vaccination was 18 years and older, but in July 2021, the indication was expanded to include children 12 years and older, as the efficacy of the vaccine in children 12 years and older was recognized. In addition, the effectiveness of the third vaccination has been recognized overseas, and a third vaccination is planned in Japan as well.

Adverse reactions to the COVID-19 vaccine have been reported frequently around the world and are the subject of ongoing discussion. BNT162b2 was the first COVID-19 vaccine licensed in the world, and reports of various adverse reactions have accumulated since its relatively early stages. The main adverse reactions are injection site reactions, fatigue, headache, and fever. In rare cases, serious side effects such as anaphylactic shock and myocarditis have been reported. However, there is still limited data on which vaccine recipients are most likely to have adverse reactions. Hoffmann et al. stated that adverse reactions in vaccinated persons who received the first dose of BNT162b2 in Germany were less common in persons over 80 years of age and in males [22]. From Korea, Bae et al. reported that there was no sex difference in adverse reactions in vaccinated subjects who received the first dose of BNT162b2, but the incidence was higher in younger age groups [23], and Lee et al. reported that the frequency of adverse reactions in vaccinated subjects who received the second dose of BNT162b2 was significantly higher in females and that the incidence was lower in older age groups [24].

In this study, we first explored the differences in adverse reactions after the first and second vaccination. As a result, the differences between the first and second vaccination were significant for many adverse reactions, and it was apparent that injection site reactions were more severe after the first vaccination, and fatigue, chills, fever, arthralgia, myalgia, headache, and nausea after the second vaccination. This result may be similar to previous reports that adverse reactions after the second vaccination are more likely to be severe [25]. Logistic regression analysis showed that for most of the adverse reactions, the incidence of adverse reactions was significantly higher in younger people. Similarly, females had a significantly higher incidence of adverse reactions than males. As for the severity of adverse reactions: the younger the vaccinated person, the more severe the adverse reaction(s), after both the first and second vaccination. This result seems to support reports of the existence of age and sex differences in response to the vaccine [26], and previous reports that the COVID-19 vaccine has a stronger immune response in females than in males and in young people than in the elderly [22]. This is probably due to the fact that antibody response to the vaccine is associated with sex hormone levels in both males and females, with females being more affected, as reported in the influenza H1N1 vaccine [27]. In recent study of RNA-sequencing data suggested that some gene expression was a promising target for association with gender-related adverse vaccine events [28]. Also, age-related result could be explained by immunosenescence which is a series of age-related changes in immune system with changes of the cells of the immune system, the soluble molecules that guide the maintenance and function of the immune system, and the lymphoid organs that coordinate both the maintenance of lymphocytes and the initiation of immune responses [29]. Although efficacy was not verified in this study, Vassilaki et al. reported higher antibody titers in females and younger vaccinated recipients [30]. In addition, a systematic review reported potential sex-related differences in efficacy and safety [31]. Therefore, it is possible that lower doses of vaccination in females and young people may provide equivalent immunity with fewer side effects. As for the medical history, it is widely known that having co-morbidities lead to high risk of developing severe conditions or high mortality rate by COVID-19 infection [32]. On the other hand, to our best knowledge, there is no evidence about the relationship between medical history and adverse effect of BNT162b2. Therefore, we exploratively included medical history as an independent variable in the analysis in this study.

This study has four limitations. The first is that the results of this study were based on a single institution in Japan, which may be affected by racial differences. Since racial differences in immunity have been reported in the past [33], and the efficacy of COVID-19 vaccine is also reported to possibly be influenced by race [34], it cannot be said that the results of this study are necessarily applicable to other races. However, since there are similar reports from other regions with fewer Asians, it is quite possible that the results of this study apply to other races as well [22]. The second is selection bias. Because the questionnaire was anonymous, it was impossible to compare the study subjects with the vaccinated staffs who were decided not to participate in this study. It is possible that some selection bias were exist with the decision to participate in this study. The third is that the adverse reactions in this study were self-reported assessments of severity. Unlike the physician's judgment of severity, this was a subjective evaluation by the vaccine recipient, making it difficult to compare with previous reports of adverse reactions to COVID-19 vaccines. However, as the importance of subjective evaluation in clinical trials has been recognized in recent years, we believe that this data is important. The fourth is sample size. Because this study is an exploratory study to identify factors that influence adverse reactions to vaccines, it is necessary to design a study that takes into account a strong hypothesis-testing analysis method that includes potential interactions and a sample size that can detect rare adverse events as independent variables.

After spreading around the world, COVID-19 has been creating waves of infection because the virus has repeatedly mutated, so that there is no prospect of ending the pandemic in the near future. The COVID-19 vaccine is one of the most powerful means of preventing infection, but antibody titers have been shown to decline over time. In addition, since there have been case reports of infection in vaccinated persons who received two doses of the vaccine, it is considered necessary to repeat the vaccination in the future, just as with the influenza vaccine. Therefore, as the results of this study indicate, it is necessary to continue to promote vaccination while also attending to the relatively strong adverse reactions in females and young people.

## Conclusion

This study revealed that adverse reactions to BNT162b2 are more frequent and more intense in females and young people. Therefore, as the results of this study indicate, it is necessary to continue to promote vaccination while acknowledging that females and younger vaccine recipients may possibly experience a higher incidence and/or greater severity of a range of typically transient, flu-like adverse reactions examined in our study. In the future, we hope that data from the third and subsequent vaccinations in Japan and from other countries will provide stronger evidence of differences in efficacy and adverse reactions depending on patient profiles. We also hope that this will lead to the development of more effective vaccinations. Additionally, the proportion of Grade 3 serious adverse reactions due to the vaccine were very low in this study. As COVID-19 vaccination is promoted worldwide, it is expected that the severity and incidence of adverse reactions will change with multiple doses or cross-vaccination with other types of vaccines. It is expected that further research on adverse reactions due to vaccines will be promoted in the future.

## Data Availability

All data generated or analyzed during this study are included in this published article.

## Abbreviations

COVID-19: Coronavirus disease 2019
SARS-CoV-2: Severe acute respiratory syndrome coronavirus 2
MHRA: Medicines and Healthcare products Regulatory Agency
NIAID: National Institute of Allergy and Infectious Diseases
CTCAE: Common Terminology Criteria for Adverse Events
OR: Odds ratio
CI: Confidence interval
